# The Medicine Success fund: financial support for widening participation medical students improves wellbeing and increases sense of belonging

**DOI:** 10.3389/fmed.2026.1887634

**Published:** 2026-09-15

**Authors:** Brigit McWade, Sarah Marsden, Charlotte Ross, Amina Iqbal

**Affiliations:** Lancaster Medical School, Faculty of Health and Medicine, Lancaster University, Lancaster, United Kingdom

**Keywords:** health profession education, inequity and social justice, medical education, social mobility, wealth justice, widening access and participation, belonging

## Abstract

**Background:**

UK medicine is an elite profession engaging in widening participation (WP) efforts to diversify the workforce so as to better represent the society it serves, and thus, enhance patient care. Despite years of sector-wide efforts, medical students and doctors from disadvantaged socio-economic backgrounds remain minoritised in medicine. WP activities primarily focus on increasing access to medical degrees, while widening success for WP students once at university is underexplored or addressed. Socio-economic disadvantage shapes the experiences of WP medical students, who face unique financial and social barriers on their journey into medicine. These include financial pressures that affect their mental health, sense of belonging, academic performance and progression.

**Innovation:**

Designed and delivered in response to our WP students’ experiences and underpinned by an ethos of wealth justice, Lancaster Medical School’s Medicine Success project offers specific financial support to eligible WP students for the hidden costs associated with studying a medical degree. Its purpose is to mitigate structural inequities embedded within the medical curriculum and higher education funding contexts, supporting the mental health and sense of belonging of WP students, together with diversifying the profession and improving patient care.

**Approach:**

This community case study describes the rationale, context, design, and delivery of Medicine Success. It reports findings from a thematic analysis of qualitative student evaluations of the project over five academic years (2019–2024), reflecting on the impact and wider policy implications of this work.

**Conclusion:**

Analysis of Medicine Success student evaluations demonstrate that even minimal financial support for WP students reduces stress and increases belonging. The cognitive and emotional impact of financial scarcity on academic performance and student participation should not be underestimated. The core design values of Medicine Success may be transferrable to other medical schools. Curriculum reviews that incorporate a wealth justice perspective, and changes to maintenance funding for medical students are also required.

## 1 Introduction: description of the nature of the problem being addressed and rationale for the proposed innovation

In the UK, medicine is an elite profession, historically dominated by white, middle or upper-class men, often with familial ties to the occupation. In 2009, the British Medical Association (BMA) asserted that “doctors should be as representative as possible of the society they serve” to enhance healthcare and reduce health inequalities (1). The aim of achieving this diversification links workforce representation directly to improving national health outcomes.

Widening Participation (WP) is a sector-wide strategy aimed at supporting historically underrepresented home-domiciled students into higher education (HE), including people from low-income backgrounds; those who attended an under-performing school, or live in an area of low participation in HE; people from minoritised ethnicities; disabled people; care-experienced people; young carers; and, other disadvantaged groups (2). All HE institutions must have a WP strategy, known as an *Action and Participation Plan* (APP) linked to funding from the *Office for Students* (OfS), incorporating evaluation to track progress.

Despite decades of WP policies and practice, research shows that inequality of access to HE has persisted and British class inequalities remain largely unchanged (3–6). Medicine also remains “one of the most inaccessible professions” for those from disadvantaged socio-economic backgrounds (7–9). Only 5% of medical school entrants come from low-income households, with most applicants coming a tiny proportion of schools, the majority of which are selective (use entrance exams, and/or tuition fees to select their pupils) (9–15). In 2026, Secretary of State for Health and Social Care, Wes Streeting MP, announced measures to widen access to healthcare professions further, stating: “We’re determined to break the class ceiling in the [National Health Service] so that our professions are elite, not elitist” (16).

While widening access is crucial, a less investigated area of WP is support for disadvantaged students once at medical school (17). Data shows an attainment gap for racially minoritised medical students and, as there is an overlap between ethnicity and socio-economic background, differential attainment is both an equality and WP issue (18). The evidence regarding causes of differential attainment is that it does not arise from an individual deficit, but rather the learning environment, curriculum, and sense of belonging (19, 20). Various initiatives, such as gateway programmes (21–23) and peer-mentoring (24–27) aim to address these barriers but focus on assimilation into established systems rather than systemic change for participation.

In 2017/2018, members of Lancaster Medical School (LMS) took a systemic approach to WP by investigating the financial and other barriers to success and progression experienced by our medical students. This led to the Medicine Success, funded by Lancaster University’s (LU) Widening Participation Action Group (WPAG) to deliver projects in line with its APP, with money from the OfS. Medicine Success offers specific financial support for the hidden costs associated with a medical degree: buying a stethoscope and professional attire and paying for a compulsory elective. Between 2018–2024, Medicine Success provided funding to 302 individual medical students, with 56% of them receiving funds during more than 1 year of study. It has funding to continue until at least 2029.

Medicine Success is iteratively developed through yearly student evaluation and grounded in an understanding of how financial inequities impede the ability of already disadvantaged students to learn, to feel well, to believe that they belong at medical school and the medical profession, or to complete their degree. It is a unique and distinct project, underpinned by a core value of wealth justice, in which we seek to preserve student dignity and agency. Rather than treating money as something we do or do not have, Medicine Success recognises money as a relationship that shapes our social, emotional and mental lives, and determines the kind of learning experiences we have access to.

This paper draws on thematic analysis of student evaluations of the project collected over a period of 5 years (2019–2024), which confirms that this support (i) *reduces stress* arising from financial scarcity, freeing up cognitive bandwidth and time to focus on studies and make the most of the learning opportunities available; (ii) *increases belonging* and self-worth, and reduces the privilege gap between peers.

## 2 Context: setting and population in which the innovation occurs

### 2.1 National context

Studying medicine in the UK presents a unique set of challenges, some of which are about material resources. A medical degree requires a minimum of 5 years to complete, potentially extended by a foundation year, intercalation, or resits. It involves longer term times than non-professional degrees, and a demanding workload, with an increasing emphasis on placements in various clinical settings over time. However, scheduling for these placements is often complex and may be poorly communicated, making it challenging for students to manage travel and accommodation costs, and accommodate any paid work (if permitted by their school). Additionally, the compulsory elective requires self-organisation, disadvantaging those with limited financial or social resources, while optional career-enhancing opportunities are often unaffordable for low-income students.

Despite these differences to other degrees, medical students do not receive additional funding for maintenance from *Student Finance England* (SFE). Minimal help with maintenance comes from the NHS bursary during their final year of study, and English home-domiciled medical students receive the least funding (28). In 2024, final year medical students were eligible for a maximum maintenance funds^1^ of £5,618 for 40 weeks.^2^ The Save the Student! National Student Money Survey 2024 found that students studying in the North West of England’s (for example) monthly living costs are c. £1085 (29). For a 5th year medical student with a longer academic year, this equates to £10,850 per annum. The maintenance funding available therefore had a shortfall of £5,232. If we compare it to the Minimum Income Standard for students (£18,632 a year outside London), which is the minimum need to thrive rather than just survive, the deficit increases to £12,174 (30).

Minimal medical education research describes the financial challenges faced by medical students, how these particularly affect WP students, how inadequate current student finance systems are, and how these result in substantial debt upon graduation (31, 32). Most discussion is led by students (17, 33). In 2022, the #LiveableNHSBursary student-led campaign highlighted the inadequate NHS bursary for WP students, revealing that financial constraints lead to lower exam marks and subsequently lower rankings for Foundation Year training compared to wealthier peers^3^ (33–36). This attainment disparity adversely affects their career prospects and earning potential. The British Medical Association (BMA) student group found that 90% of all medical students do not receive enough funding to cover everyday costs, and half had considered leaving their course and medicine. They emphasised the distress caused by financial insecurity and launched the #FixOurFunding campaign in 2024, advocating for full student finance maintenance funding for all study years (28, 37). Research shows that qualified doctors from lower socio-economic backgrounds, with an average class pay gap of £3,640, thus financial inequities experienced during training are not a temporary cost for a lucrative career (38). Yet, despite ongoing commitments to widening access by consecutive governments, changes have yet to be made to address the financial barriers inherent in the structure of medical degrees.

### 2.2 Local context

LMS was founded in 2006 at LU in the North-West of England. Over the period 2019–2024, total student numbers increased from 345 to 802. Around one third of its students fulfil its WP criteria at entry or are identified as doing so during their degree. Table 1 lists the WP criteria specific to LMS at application (39).

**Table 1:** Lancaster Medical School’s WP admissions criteria.

Medical school WP criteria
One of the following criteria
You are or have been in local authority care for 3 months or more, or considered Care Experienced, including residing within a secure or children’s care home, foster care, and kinship care.
You are a refugee.
Or, two or more of the following criteria
You live in a neighborhood with low participation in higher education or an area of disadvantage (check the Index of Multiple Deprivation, Scottish Index of Multiple Deprivation or the Office for Students’ postcode look up tool).
You live in a low-income household, demonstrated by one of the following: Parent/guardian receives means-tested benefits (e.g., Universal Credit, Job Seekers Allowance) You receive or are eligible for the 16–19 bursary or equivalent You receive or are eligible for free school meals
You attended a non-selective state school for your GCSEs with an Attainment 8 score below the national average. This is calculated by the Admissions team based on recent Department for Education data.
You attend(ed) a non-selective state school/college for you’re a levels with results below the national average. This is calculated by the Admissions team based on recent Department for Education data.
Your parent(s) did not attend university or attain a higher education qualification, either in the UK or abroad, apart from as a mature student over the age of 25 and did not graduate as either a doctor or dentist.
You are a young carer to a parent or sibling.

In 2017/2018, LMS’s then Lead for WP, Nicola Burns, secured funding for a needs analysis into the financial and other barriers to learning experienced by LMS medical students. The needs analysis confirmed what had already been reported by the Medical Schools Council (MSC) in 2014 about the additional challenges faced by all medical students, as described above (40). These data supported our first successful application for funding for *Medicine Success*, a tailored version of LU’s Student Access and Success (SAS) Programme. The lead author of this paper has been solely responsible for the project since 2019.

## 3 Key programmatic elements

### 3.1 Practical aspects

#### 3.1.1 Funding the project

The project is funded with OfS monies for WP, awarded via the institution’s WP action group. As student numbers increased, extra funds were secured. To begin with, we had to apply each year to renew and increase the amount awarded. After 3 years of evidence that the project was effective, we secured 3 years of funding, which freed up time to focus on improvements and expansion of the project. In 2025, we were awarded 4 years of funding. The amount applied for was estimated on the school’s records of how many we students we have and the proportion of WP students per intake. Table 2 shows the amounts received and distributed each year.

**Table 2:** Funding received for MS each year.

Academic year	Amount received
2019–2020	£19,221
2020–2021	£24,430
2021–2022	£31,826.50
2022–2023	£48,749.25
2023–2024	£55,826

#### 3.1.2 Funds available

To date, the specific elements of the Medicine Success Programme package have included:

1. *Stethoscope fund*: an essential tool for medical training. All first-year medical students are required to buy one at a cost of £80–£90.
2. *Professional attire fund*: This fund helps WP students purchase professional attire required throughout their medical studies. Medical students must follow a strict dress code from Year 1 onwards, which can present a significant financial burden. Students are given ~£200 in the first year to build a capsule work wardrobe, and then ~£100 for the following years of study to refresh it. The most popular item bought with these funds are a good pair of comfortable and hardwearing shoes.
3. *Elective fund*: This bursary supports Year 4 WP students undertaking their compulsory elective placement. Electives are a core part of the curriculum, allowing students to gain experience in different clinical settings, including international and low-resource environments. Despite being compulsory, electives are self-funded. Funds are awarded according to need, with a mean average of £650 per student. This fund enables students to do the elective they want to do, not just the one they can afford.

Student evaluations found that recipients were spending their funding for stethoscope and professional attire on a wider range of items including placement travel costs, subscriptions to online revision resources, scrubs, appropriate shoes for placement, professional attire specifically for their elective, and travel to conferences. Since 2024, the stethoscope and professional attire funds have thus been grouped together as the *Medicine Success* fund. Suggestions of how to spend are offered but not enforced. The Elective remains a fund for electives only. All evaluations ask what the student spent the money on for the purposes of identifying financial barriers experienced across the cohort, rather than identifying individual students’ misappropriation of funds (see section 3.2.2).

#### 3.1.3 How applications are assessed and awarded

Applications are made via an online LU Qualtrics secure survey platform to which only the Medicine Success lead has access to. Once the closing date has passed, applications are downloaded and data regarding WP criteria is verified using university records. Since 2024, to ensure those most in need receive funds, a weighted scoring system was introduced (Table 3). Applicants need to score at least one point to be awarded the funding.

**Table 3:** Eligibility criteria for *medicine success* and rationale for weighted scoring system.

Criterion	Points	Reason
You received a contextually lowered offer when they applied for your degree at LMS	0	The criterion for receiving a CLO is the same as some of the criteria for Medicine Success, so to avoid double counting, this receives no points
You completed the LMS Foundation Year or Gateway Year before commencing the MBChB	0.5	While acceptance onto the foundation or gateway year involves meeting some of the same criteria for Medicine Success, it also extends the length of the degree and therefore the costs incurred.
You are from an area of multiple deprivation as measured through IMD, NISA or WIMD	0.5	Coming from a deprived area does not necessarily equate to low-income or financial scarcity
You are from a low-income background	2	Students from a low-income background are the priority group for these funds
You are a mature student (commenced the degree after the age of 21 years old)	1	Mature students may have to pay their own tuition fees, or have debts from previous higher education degrees
You are disabled; this includes those with mental health diagnoses and neurodivergent students	1	Evidence clearly demonstrates that life is more expensive for disabled people
You are or have been a refugee	1	Displacement is often accompanied by trauma and loss of assets, and restrictions on welfare and working.
You are a carer or a parent	1.5	Having social and financial dependents incurs greater costs
You are a care leaver or care experienced	1.5	Financial challenges are widely recognised as a barrier to participation for care leavers and care experienced university students
You are estranged from your family	2	As above
You are first in your family to attend university	0.5	This does not necessarily equate to financial scarcity, but it may mean that your family does not have the knowledge or desire to support their children through university
You belong to a racially minoritised group	0.5	This does not necessarily equate to financial scarcity and cannot be counted on its own as a reason for financial support.

Once eligible applicants are identified, awards are made. There is flexibility for over and under spend of any pot to be redistributed where there are more or less applicants than funds. All applicants are notified of the outcome and are invited to complete an evaluation of the funding to feed into future applications, including those who were not successful. Our professional services team support the dissemination of the funds.

#### 3.1.4 Evaluation

Each year, recipients of MS funds were emailed and asked to complete an evaluation on the LU Qualtrics secure survey platform. The survey contained a mixture of quantitative Likert scale numerical evaluative and free-text qualitative questions. Between 2019–2024, numbers of respondents varied between 44–140 over the 5 years, with a mean response rate of 62%. The response rate was reduced due to staffing issues that disrupted the project in 2021–2022 resulting in a much lower number than usual (37% compared to a high of 79% and a low of 59%).

### 3.2 Core design values of Medicine Success

In this section, we outline the ethos and core values of *Medicine Success*, rooted in evidence relating to wealth and welfare and our evaluations of the project.

#### 3.2.1 Wealth justice

Research across multiple disciplines demonstrate the impact of impoverishment on wellbeing and cognitive function. It has been shown that people with scarce financial resources are regularly, persistently and spontaneously provoked to think about money “even when money has not been mentioned,” (re)shaping the way they connect different things in their minds (41). Further, financial aspects of our lives are influenced by past trauma and can also prevent recovery from trauma at the same time (42). Impoverishment transforms lived experience of the world, and in turn shapes thoughts and feelings in ways that last long after money might become more plentiful. Meaningful WP work must therefore reject the idea that higher education degrees lead to accruing wealth and recognise that it is sufficient to increase places at medical school for “non-traditional” students. Thus, wealth justice is a tenet of Medicine Success recognising that a liveable income is one of the central “material, social or emotional conditions that are necessary to make the process of education itself possible” (43).

The current funding landscape for medical students is an example of “financial trauma,” defined by Chloe McKenzie as “the cumulative effect of being required to experience economic violence, financial abuse, financial shaming, and/or (chronic) financial stress to attain or sustain material safety” (44). We place our students’ experiences of financial scarcity in context, acknowledging how this “trauma is relevant to a person’s socioeconomic experiences and wealth-building capability” (45). This reframes WP and social mobility as a social and structural intervention, rather than an individualised one.

#### 3.2.2 Trust, dignity and agency

A core value embedded in Medicine Success is to recognise that our students are adults with agency and dignity, who deserve respect and support. Current trends in welfare systems lean towards a punitive approach that requires the investment of substantial emotional and time resources from the applicant and intense scrutiny as to their deservingness (46, 47). Medical students are time poor, especially those who have to work part-time to support their studies, or have caring responsibilities (48). The school is responsible for their wellbeing and ability to make the most of the opportunities available to them during their degree. To avoid reproducing inequities, the Medicine Success application process is short, quick, simple, and extra hurdles to prove eligibility, such as a personal statement, references or financial statements are not required. This signals to our students that we trust them and that they are no lesser than their peers who may be more financially comfortable, destigmatising asking for the support they are entitled to. As one student reflects:

“This funding process was well-advertised, straightforward, and dignified, unlike many other sources that can feel stigmatising or require proving one’s “worthiness” for funding (which makes you feel both disadvantaged and unduly entitled)” (4th year, 2023).

Secondly, students are given the money directly into their bank account, they are permitted to spend it on what they deem important and are not required to provide receipts. This eschews judgement that those experiencing financial scarcity are responsible for their own poverty because they have poor money management skills (49). It recognises that inequities in income and wealth are socially constructed and not the result of laziness or frivolity. It reinforces the message that we trust our students and respect their agency and autonomy.

Student evaluations repeatedly show that Medicine Success makes students from underprivileged backgrounds feel that that they are being recognised, supported and cared for by the medical school:

“The funding makes a huge difference, as firstly it is a big financial aid, but secondly it also makes medical students feel that they are cared for by their medical school and it reassures them that it is okay for some of us to have struggles, and that we will be helped out” (1st year, 2020).

The funds motivate students to keep going when they may otherwise be struggling, knowing that someone is advocating for them and that funding will be available in future years. They are made to feel deserving of being a medical student, which alleviates some of the imposter syndrome that is generated by intense and highly competitive courses such as medicine.

#### 3.2.3 Listening and learning

The design and delivery of Medicine Success is rooted in the experiences of WP students. Each year, recipients provide feedback through an online qualitative survey, enabling iterative improvement of the programme and highlighting unaddressed issues. This student-centred approach enhances our understanding of the financial challenges faced by university medical students, particularly concerning wealth-based exclusion.

We identified critical points at the beginning and end of the medical degree. The initial costs of attending university can be high due to moving expenses and one-off purchases such as essential domestic items and learning equipment. Consequently, unexpected costs, such as acquiring a stethoscope just before term, can create a stressful start for new medicine students. Medicine Success supports first-year students as they manage budgets for the first time while balancing new life skills and peer relationships. Additionally, the programme eases financial pressures in students’ final year, addressing issues related to reduced funding and increased placement demands.

While the hidden costs of a medical degree may seem minimal, they can significantly stretch budgets without support. Access to Medicine Success funding for essentials can reduce the necessity for part-time work, allowing students to focus more on their studies. Recipients have noted that this financial assistance provides them with more time and cognitive capacity to excel academically, contributing to a more equitable environment with wealthier peers.

WP medical students also recognise the inequalities they face compared to their more privileged counterparts. They articulate how Medicine Success funding helps bridge this privilege gap, enhancing their sense of self-worth and belonging, thereby supporting their academic success and future careers. The funding is crucial as it enables them to participate proudly and equally in their education. For example, students have highlighted the impact of clothing on perceptions of professionalism and their ability to engage in practical learning during clinical placements. Financial support allows them to purchase appropriate attire and essential tools like stethoscopes, facilitating skill development outside the classroom. This access reduces disadvantages, ensuring that all students can practice essential skills without delay.

#### 3.2.4 Amplify and advocate

The rich evaluation data is used beyond the project to educate all members of the medical school as to the realities of studying while poor, including annual reports to committees and presentations to the wider community as part of Continuing Professional Development (CPD) events. Findings have helped instigate curriculum change and develop additional initiatives to support WP students access all the opportunities available to them during their studies, for example changes to the travel policy for clinical placements and provision of free subscription codes to revision resources.

These data also support the need for systemic change to curricula and funding for medical degrees if WP is to be meaningfully achieved. In publishing findings from our project, we seek to influence national higher education and medical education funding. In many ways, although highly prized by our students, the project is an insufficient sticking plaster on a much larger issue with the way medical degrees are financed and the structure and requirements of the medical curriculum. As part of the project, we seek to change the narrative around WP students away from individual deficit to advocating for structural change, hopefully making Medicine Success a stepping stone to meaningfully diversifying the medical profession.

## 4 Discussion: practical implications and lessons learned for future applications

The core values and ethos of Medicine Success is transferable to all medical schools in the UK, and internationally, whether WP is a national concern or not. To effectively support students, medical schools must be familiar with WP discourse, or cognate debates, both in HE and ME in their context. They must familiarise themselves with policies and funds available to support the diversification of graduates and medicine.

However, adopting a wealth justice approach to social mobility will likely go against current dominant narratives. Social mobility is a problematic term; it requires individual people to increase their position in an established hierarchy that is itself integral to maintaining socioeconomic inequality. Widening access to HE relies on the narrative of meritocracy, in which any failure to achieve success cannot be blamed on those opening doors, but on those who fail to achieve their potential once they come in. The logic proceeds thus: “if those from different backgrounds have fair access to the most desirable jobs and highest incomes, any inequality in outcome that follows is acceptable, and possibly even desirable (as higher rewards should motivate the most capable to seek the most prized jobs)” (50). Medicine Success pushes back at this discourse, and grounded in student lived experiences, reframes money as a social relationship integral to the learning environment, sense of belonging, and success.

In the UK, each school exists in different WP funding contexts, as every institution has their own individual authored APP and distributes the OfS money awarded to them differently. Each school also has its own group of students with specific characteristics, curriculum structure, culture, geographical location, and so forth. It is thus important that each school establish need, and the specificities of that need, according to their own peculiarities, and apply for funding where appropriate to take such a project forward.

Ideally, the application process would be removed entirely, and on entry each student would be assessed as to need once and then awarded the money throughout the degree without any further input from them. This is difficult to achieve as, from our experience reviewing applications to our medical schools, students are sometimes reluctant to identify as WP because of the stigma attached, or because they do not recognise themselves in that label. Related research on welfare support in the UK also finds uptake of social security payments are affected by feelings of shame and stigma meaning “millions of pounds are left unclaimed annually in benefit” due to people feeling that they do not deserve help (51). Moreover, circumstances can change, students are diagnosed with disabilities while at university, become estranged, become parents or carers, their parents become ill, or lose income and so on. Consequently, the ever changing and elusive classification of students makes it difficult.

## 5 Conceptual or methodological constraints

We draw on available robust evidence and theoretical work exploring the relationship between poverty and learning, and the lived experiences of medical students with limited financial means. We have yet to be able to measure impact on retention, attainment and progression. As with many WP initiatives, evidence remains qualitative, and it is almost impossible to isolate the application of a social activity on a discrete outcome. This is something we are working on but expect it to take some time and methodological innovation to achieve.

## Data Availability

The datasets presented in this article are not readily available because as this is a secondary dataset, collected in the first instance for internal quality monitoring, without informed consent from participants for sharing, we cannot make it available. Requests to access the datasets should be directed to BM b.mcwade@lancaster.ac.uk.

## Footnotes

1 A combination of a non-means tested loan, and a non-means tested grant.

2 We list figures for 2024, as this relates to the data we draw on throughout the paper. There have been no major changes since 2024, beyond a small increase aligned with inflation.

3 Ranking has now been removed from FY placements, but the problem is far from resolved. It is not within the scope of this paper to address this.
